# Loss of thyroid gland circadian PER2 rhythmicity in aged mice and its potential association with thyroid cancer development

**DOI:** 10.1038/s41419-022-05342-2

**Published:** 2022-10-26

**Authors:** Junguee Lee, Hae Joung Sul, Hyunsu Choi, Dong Hyun Oh, Minho Shong

**Affiliations:** 1 Department of Pathology, Daejeon St. Mary’s Hospital, College of Medicine, The Catholic University of Korea, Seoul, Republic of Korea; 2 Clinical Research Institute, Daejeon St. Mary’s Hospital, College of Medicine, The Catholic University of Korea, Daejeon, Republic of Korea; 3 Department of Radiology, Konyang University Hospital, Daejeon, Republic of Korea; 4 Department of Internal Medicine, Chungnam National University School of Medicine, Daejeon, Republic of Korea

**Keywords:** Circadian rhythms, Oncogenesis, Senescence

## Abstract

Molecular clocks operate in peripheral tissues, including endocrine glands, and play important regulatory roles in this context. However, potential age-related changes in the expression rhythmicity of clock genes and the effects of these changes on the thyroid gland remain unknown. In the present study, we evaluated the expression rhythmicity of peripheral thyroid clock genes in aged mice using RNA-seq transcriptomic analysis in young (3.5-month) versus aged (20-month) mice. In addition, we determined the cellular effects of silencing of PER2, a major clock gene regulator, in human thyroid cell lines. Kyoto Encyclopedia of Genes and Genomes (KEGG) pathway enrichment analysis revealed that differentially expressed genes (DEGs) in the thyroid glands of aged mice were involved in mitogen-activated protein kinase (MAPK) signaling, chemokine signaling, circadian entrainment, PI3K/AKT signaling, and Apelin signaling. The expression of circadian clock genes *Arntl/Bmal1* was significantly downregulated in thyroid glands of aged mice, whereas the expression of genes involved in regulation of cell proliferation, migration, and tumorigenesis was upregulated. Peripheral thyroid clock genes, particularly *Per* mRNA and PER2 protein, were downregulated in the thyroid glands of aged mice, and circadian oscillation of these genes was declined. Knockdown of the circadian clock gene *PER2* in human thyroid follicular cells induced AP-1 activity via JNK MAPK signaling activation, which increased cell proliferation. Furthermore, the aging-related loss of *PER2* circadian oscillation activated the AP-1 transcription factor via the JNK MAPK pathway, which could contribute to thyroid hyperplasia, a common age-related condition.

## Introduction

The mammalian circadian clock mechanism is comprised of circadian clock genes, whose expression oscillates with the circadian rhythm in the brain and in peripheral organs. The central circadian clock is located in the hypothalamic suprachiasmatic nucleus (SCN) [1]. Cellular peripheral clocks, regulated by expression of clock genes, operate in every organ of the body. These endogenous circadian oscillators influence gene expression in a tissue-specific manner and are responsible for coordinating circadian regulation of the tissue’s physiologic functions [2]. Mammalian clock genes form a transcription-translation feedback loop (TTFL) consisting of the transcriptional activators Circadian locomotor output cycles kaput (CLOCK) and Aryl hydrocarbon receptor nuclear translocator-like (ARNTL)/BMAL1, and the transcriptional repressors Period-1 (PER1), PER2, PER3, Cryptochrome-1 (CRY1), and CRY2 [3]. The CLOCK:BMAL1 heterodimer binds to E-boxes in the promoter sequences of *Cry* and *Per* genes, activating their transcription. Subsequently, increased CRY protein levels inhibit gene transcription of CLOCK:BMAL1 at the promoter and then block E-box binding of the CLOCK:BMAL1 heterodimer. Accumulated PER and CRY proteins form a PER-CRY heterodimer, which inhibits transcriptional activation of downstream genes by reducing CLOCK:BMAL1 binding to the E-box, generating circadian oscillation [4, 5]. In humans and rodents, appropriate oscillation of the central circadian clock is impaired with aging [6, 7]. Expression of clock genes and appropriate oscillation of their expression is also impaired by aging in peripheral tissues [6, 7]. These aging-associated rhythmic declines of peripheral clock gene expression could have implications for aging-associated diseases, such as metabolic diseases and cancers [8, 9].

Aging is a driving factor of functional, structural, and molecular changes in the thyroid gland. Thyroid hypofunction is common in the aged population [10, 11]. Moreover, the thyroid gland undergoes histological changes during aging characterized by the appearance of papillary and glandular hyperplasia of thyroid follicles [11]. Transcriptomic analyses of the aging human thyroid have revealed mitochondrial and proteasomal dysfunction, defective cell differentiation, and activation of autoimmune processes [12]. The importance of these aging phenotypes of the thyroid gland is increasingly recognized, as aging-associated thyroid dysfunction could affect the risks of cardiovascular disease, cognitive impairment, depression, and longevity [13]. Further, the incidence of thyroid cancer increases with aging, as does its poor prognosis [14]. Many studies have revealed a statistically significantly higher rate of anaplastic thyroid cancer and advanced cancer stages and more frequent distant metastasis in elderly thyroid patients than in young thyroid cancer patients [15].

Thyroid function is regulated in part by the central circadian clock. Fahrenkrug et al. demonstrated that the circadian rhythm of thyroid hormone release from the thyroid gland is regulated by rhythmic SCN-induced thyroid stimulating hormone (TSH) release from the pituitary gland rather than by the peripheral thyroid clock [16]. However, Mannic et al. demonstrated that disruption of peripheral thyroid clock in the human thyroid plays a potential role in malignant transformation of thyroid nodules [17]. However, local regulation and potential disruption of the thyroid peripheral circadian clock during the aging process remain incompletely understood.

In the present study, we aimed to determine aging-related thyroid changes in mice using RNA-seq analysis. Furthermore, we elucidated histological change-related transcriptional signatures in aged murine thyroids and subsequently explored the relationship between aging and attenuation of the thyroid circadian clock machinery.

## Materials and methods

### Mice and ethics statement

C57BL/6 male mice were fed a normal chow diet and housed since birth in a 12 h light/12 h dark cycle (7:00 am lights turn-on, 7:00 pm lights turn-off). Mice were classified into three groups according to age: Group 1, 3.5-month of age (mature adult, *n* = 14), Group 2, 10-month of age (middle-aged, *n* = 3), and Group 3, 20-month of age (aged, *n* = 15). Thyroid glands extracted from three mice per group were used for RNA sequencing analysis.

To obtain mouse thyroid and liver sample for circadian rhythm analysis, mice were sacrificed and thyroids and livers isolated in 6 h intervals over the circadian cycle (Zeitgeber time [ZT] 0, 6, 12, and 18 h). Light is arguably the most influential zeitgeber for the entrainment of circadian rhythms. Therefore, we used light as a zeitgeber (light-on at ZT 7 h and light-off at ZT 19 h). Three mice per group were sacrificed at each timepoint. For immunoblot analysis, thyroids from 3.5-month-old (*n* = 8) and 20-month-old (*n* = 8) mice were used additionally. Two mice per group were sacrificed at each timepoint (ZT 0, 6, 12, and 18 h). Animal experiments received prior approval by the Institutional Animal Care and Use Committee of the Catholic University of Korea (approval ID, CMCDJ-AP-2019-007).

### Human thyroid follicular cells and thyroid cancer cells

Nthy-ori 3.1 (a non-transformed human follicular cell line obtained by the European Collection of Authenticated Cell Cultures) and TPC1 (a human RET/PTC rearrangement PTC cell line; from Dr. M. Takahashi, Nagoya University, Japan) were cultured in RPMI 1640 (WELGENE Inc. Republic of Korea) supplemented with 10% fetal bovine serum (HyClone, USA) and 1% Gibco penicillin/streptomycin (Thermo Fisher Scientific Inc.) at 37 °C and 5% CO_2_.

### Human thyroid tissue microarray with non-tumor and papillary carcinoma

Formalin fixation and paraffin-embedded tissue blocks of human thyroids were obtained from patients that underwent thyroidectomies between January 2002 and December 2005 at St. Mary’s Hospital, Daejeon, South Korea. Only women’s thyroids were used. The study protocol was reviewed and approved by the Institutional Review Board and all methods were conducted in accordance with the guidelines approved by Daejeon St. Mary’s Hospital, College of Medicine Catholic University of Korea (approval ID, DC20SISI0056). Paraffin tissue blocks with non-tumor thyroid and papillary thyroid carcinoma (PTC) from each patient were selected. Tissue cores of 5 mm in diameter were collected from non-tumor and PTC portions, respectively, and were rearranged in the recipient paraffin blocks.

### RNA sequencing analysis

Thyroid glands extracted from three mice per group (Group 1, 3.5 months of age; Group 2, 10 months of age; Group 3, 20 months of age) were used for RNA sequencing. Mice were sacrificed and thyroids isolated during a ZT 10-12 h. Extracted thyroid glands were sent to Macrogen Inc. (Seoul, South Korea) for RNA sequencing analysis. A TruSeq Stranded Total RNA LT Sample Prep Kit (Gold) was used as a library kit, and the NovaSeq 6000 S4 Reagent Kit was used as a reagent. DEG analysis was conducted on three comparison combinations using FPKM (Fragment per Kilobase of transcript per Million mapped reads), and 756 genes were extracted from at least one comparison combination that met |fc| ≥ 2 and independent *t*-test raw *p*-value < 0.05.

Gene annotation enrichment analysis using Gene Ontology (GO) (https://biit.cs.ut.ee/gprofiler/) and KEGG (http://www.genome.jp/kegg/) data for gene sets was performed. The GO enrichment analysis was performed using terms grouped into three categories: molecular function, biological processes, and cellular components.

### Knockdown of *PER2* using RNA interference

Cells were plated in 6-well cell plate and allowed to adhere overnight. Cells were transfected with 20 nM *PER2* siRNA (Assay ID 114920, Thermo Fisher Scientific Inc.) in Opti-MEM I medium using Lipofectamine RNAiMAX transfection reagent (Invitrogen) following the manufacturer’s instructions. Negative control siRNA containing non-specific sequences with no homologs in the human genome was also provided by Invitrogen. All experiments were performed in triplicate and repeated at least three times. The efficiency of gene knockdown was verified by RT-qPCR 48 h after transfection.

### Total RNA isolation and RT-qPCR

An Easy-BLUE Total RNA Extraction Kit (iNtRON Bio) was used to extract total RNA from cultured cells and mouse thyroid tissue. M-MLV Reverse Transcriptase and oligo-dT primers (Invitrogen) were used to synthesize complementary DNA (cDNA) from total RNA. Reverse transcription-quantitative polymerase chain reaction (RT-qPCR) was performed using an Applied Biosystems 7500 Real-Time PCR System (Thermo Fisher Scientific Inc.) with QuantiTect SYBR Green PCR Master Mix (QIAGEN). Each reaction was conducted in triplicate. The sequences of qPCR primers are listed in Supplementary Table S1. Amplification conditions were as follows: enzyme activation for 10 min at 95 °C, denaturation for 10 s at 95 °C (40 cycles), annealing at 60 °C for 30 s, and extension at 72 °C for 30 s. The fold change of individual genes was determined using the delta cycle threshold (Ct) values, and normalized to the *Gapdh* values using computer software (Life Technologies 7500 software, Foster City, CA, USA).

### Immunoblot analysis

Immunoblotting was conducted as previously described [18]. The following primary antibodies were used: PER2 (biorbyt, 1:1000), phospho-AKT (Ser473)(Cell Signaling Technology, 1:1000), phospho-p70S6 kinase (Cell Signaling Technology, 1:1000), phospho-p38 MAPK (Cell Signaling Technology, 1:1000), ERK1/2 (Cell Signaling Technology, 1:1000), phospho-ERK1/2 (Cell Signaling Technology, 1:1000), JNK (Cell Signaling Technology, 1:1000), phospho-JNK (Cell Signaling Technology, 1:1000), c-Fos (Santa Cruz, 1:1000), c-Jun (LSBio, 1:1000), and GAPDH (Abcam, 1:1000). GAPDH was used as a loading control. ImageJ gel analysis was used to quantify the densities of bands on immunoblots.

### Immunohistochemistry

Staining of formalin-fixed and paraffin-embedded murine thyroid tissue sections and human tissue microarray was conducted as previously described [19]. The primary antibody was anti-rabbit PER2 (biorbyt, 1:100) and Ki-67 (Cell Signaling Technology, 1:200).

### Cell proliferation/viability assay and scratch wound healing assay

Cell proliferation was evaluated using an MTT assay kit (Sigma-Aldrich) following the manufacturer’s instructions. Cells were transfected with si*PER2* and plated in 96-well plates 24 h later. MTT solution was added to the plated cells. Absorbance was measured 0, 60, and 90 min later in a plate reader (wavelength 595 nm) (Molecular Devices, Sunnyvale, CA, USA).

The scratch assay was used to assess cell migration. Cells were seeded in six-well plates and transfected with si*PER2*. Following formation of a confluent monolayer for 24 h, cell surfaces were vertically scratched using a p200 pipette tip. The cells were observed zero and 24 h post-scratch and images of the cells were subsequently captured using an Olympus IX71 microscope (Olympus). The wound width can be calculated as the average distance between the edges of the scratch.

### Cosinor-based rhythmometry analysis

Cosinor analysis was used to assess the circadian rhythmicity of clock genes, such as the periodicity and the distribution of changes in expression over time (CosinorJ software version 1.0) [20]. Three cosinor parameters (MESOR, amplitude, acrophase) were included in the study [21, 22].

### Statistical analysis

One-way ANOVA (IBM SPSS Statistics 22.0) was applied to evaluate whether gene expression oscillated throughout the 24 h period. Two-way ANOVA (IBM SPSS Statistics 22.0), followed by Bonferroni’s post hoc analysis, was used to evaluate the influence of time and age on gene expression. Group comparisons of categorical variables were performed using the Chi-square test (IBM SPSS Statistics 22.0). Data are presented as means ± standard error of the mean (SEM). The statistical significance of differences between two groups was determined using a Student’s *t*-test. A *p*-value < 0.05 was considered significant (*P < 0.05, **P < 0.01, ***P < 0.001).

## Results

### Gene expression-based change of the thyroid glands in 20-month-old mice

**Fig. 1:**
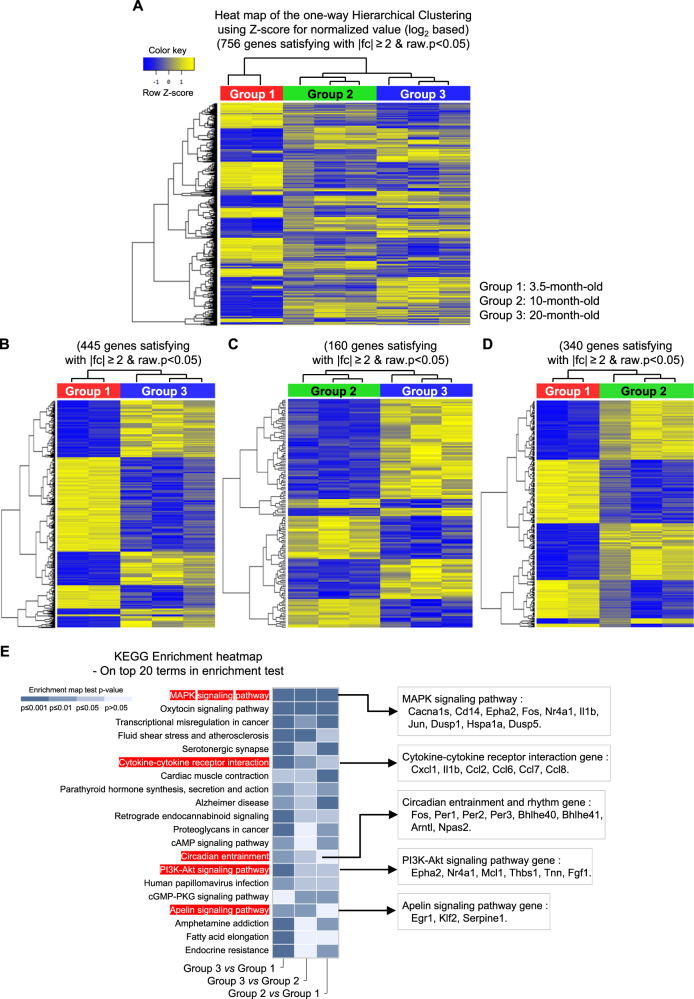
Gene expression-based change of the thyroid glands in 20-month-old mice. **A** Hierarchical clustering heatmap of differential gene expression. Heatmap of one-way hierarchical clustering using the expression for a total of 756 unigenes (rows) satisfying the arbitrary criterium of a ≥2-fold change in expression and a raw *p*-value < 0.05 in eight samples (columns). Expression levels of each unigene across samples are presented as row Z score calculated by FPKM from RNA sequencing. **B** Group 1 (3.5 months of age) and Group 3 (20 months of age) differential gene expression. **C** Group 2 (10 months of age) and Group 3 (20 months of age) differential gene expression. **D** Group 1 (3.5 months of age) and Group 2 (10 months of age) differential gene expression. **E** Heatmap of KEGG pathways enrichment scores of DEGs in each comparison combination.

To determine if gene expression changes by RNA-seq in the thyroid gland were sufficient to distinguish three age groups of mice (Group 1, 3.5-month; Group 2, 10-month; Group 3, 20-month), we performed unsupervised one-way hierarchical clustering, which revealed significant differences in gene expression between groups (Fig. 1A). The 756 genes that met the arbitrary criterium of a ≥2-fold change in expression with a raw *p*-value <0.05 are presented as a heatmap (Fig. 1A, Supplementary Fig. S1A). To identify significantly changes in gene expression in the aged murine thyroid, we then performed pairwise comparisons of the gene expression between mice of different ages (3.5- *vs*. 10-month, 3.5- *vs*. 20-month, and 10- *vs*. 20-month) (Fig. 1B–D). Hierarchical analysis of significantly altered genes revealed distinct differences in expression patterns among the three groups. The gene lists of each heatmap are provided in Supplementary Fig. S1B–S1D.

To gain insights into the functional characteristics of differentially expressed genes (DEGs) that occur during mouse thyroid aging, we used KEGG pathway enrichment analyses. KEGG pathway enrichment analysis of DEGs (≥2-fold change and raw *p*-value < 0.05) identified that the major enriched pathways of Group 3, the oldest mice in this study, were involved in MAPK signaling (*Cacna1s*, *Cd14*, *Epha2*, *Fos*, *Nr4a1*, *Il1b*, *Jun*, *Dusp1*, *Hspa1a*, and *Dusp5*), cytokine-cytokine-receptor interactions, and chemokine signaling (*Cxcl1*, *Il1b*, *Ccl2*, *Ccl6*, *Ccl7*, and *Ccl8*), circadian entrainment (*Fos*, *Per1*, *Per2*, and *Per3*), phosphatidylinositol 3 kinase-protein kinase B (PI3K-AKT) signaling (*Epha2*, *Nr4a1*, *Mcl1*, *Thbs1*, *Tnn*, and *Fgf1*), and Apelin signaling (*Egr1*, *Klf2*, and *Serpine1*) (Fig. 1E).

### Gene expression signature in aged murine thyroids

**Fig. 2:**
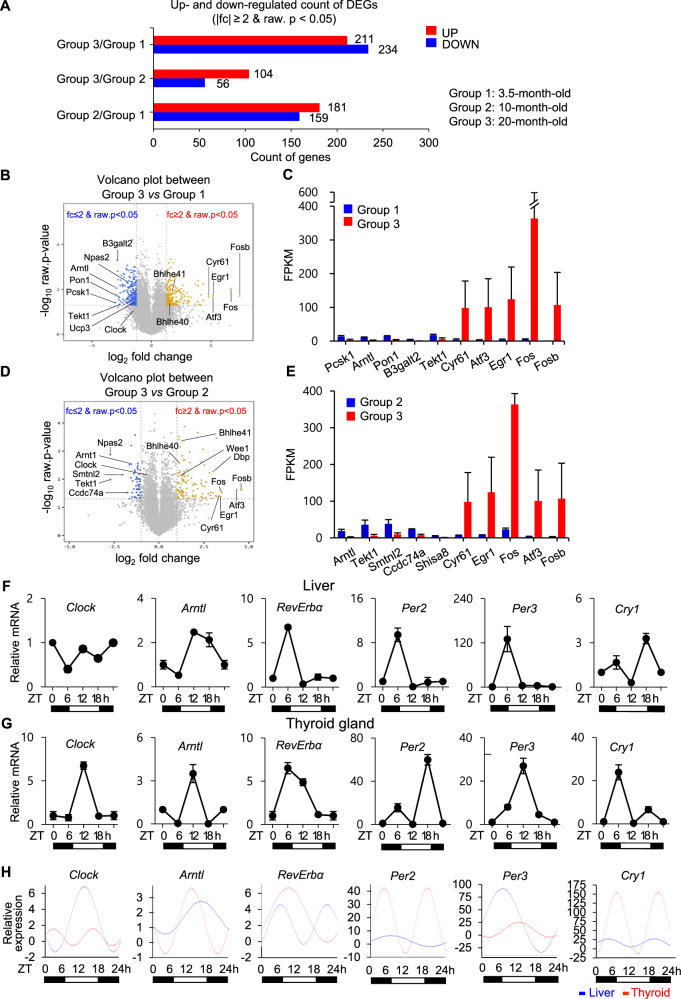
Gene expression signature in aged murine thyroids. **A** Graph representative of significant upregulated and downregulated counts of DEGs based on fold change and *p*-value in Group 3 (20 months of age) vs. Group 1 (3.5 months of age), Group 3 vs. Group 2 (10 months of age), and Group 2 vs. Group 1. The Y-axis represents up- and down-regulation of DEGs between each comparison. The X-axis represents the total number of DEGs. **B**, **D** Volcano plots represent DEGs in each comparison combination. The symbol-marked dots indicate the top five upregulated and downregulated genes and clock genes. **C**, **E** Each bar graph represents the five genes with the largest negative or positive standardized mean difference. **F**, **G** mRNA expression of peripheral circadian clocks in the murine liver and thyroid of 3.5-month-old mice at the indicated times. Transcript levels of core clock genes (*Clock*, *Arntl/Bmal1*, *Rev-erbα*, *Per2*, *Per3*, and *Cry1*) are determined by qPCR analyses. Gapdh is used as an internal control. Gene expression is analyzed in 6 h intervals (ZT 0, 6, 12, 18 h; *n* = 3 mice/timepoint). Values are expressed as relative mRNA levels of three separate experiments for each gene. **H** Cosinor-based rhythmometry analysis is used to determine the rhythmic expression of clock genes.

We searched for differentially expressed genes between the age groups that met the arbitrary criterium of a ≥2-fold change in expression with a *p*-value < 0.05. Using these criteria, we detected downregulation of 234 genes in 20- *vs*. 3.5-month-old mice, 56 genes in 20- *vs*. 10-month-old mice, and 159 genes in 10- *vs*. 3.5-month-old mice, as well as upregulation of 211 genes in 20- *vs*. 3.5-month-old mice, 104 genes in 20- *vs*. 10-month-old mice, and 181 genes in 10- *vs*. 3.5-month-old mice (Fig. 2A). Genes included in further analyses were sorted by GO annotation (Table 1). Genes associated with upregulation or downregulation of circadian entrainment and circadian rhythm and aging are listed in Table 1. Aging-related genes, and genes involved in immunity and inflammation, were upregulated in thyroid of 20-month-old mice relative to those in 3.5- or 10-month-old mice. Genes related to thyroid differentiation were only modestly altered between groups. Genes related to obesity development and lipid metabolism were downregulated in thyroid of 20-month-old mice relative to those in 3.5- or 10-month-old mice. Loss-of-function mutations of *Pcsk1*, *Cnr1*, and *Bbs10* contribute to the development of obesity-associated features including severe early-onset obesity, increased waist circumference and skin-fold thickness, and higher adiposity [23–25]. Notably, we found that genes associated with tumorigenesis and tumor progression were upregulated in thyroid of 20-month-old mice relative to those in 3.5- or 10-month-old mice.

**Table 1:** Gene expression signature in aged murine thyroids.

Gene		20 month/3.5 month	20 month/10 month	10 month/3.5 month
		Log_2_ fold change	Log_2_ fold change	Log_2_ fold change
**Circadian rhythm and entrainment genes**				
*Downregulated*				
Arntl		−5.14^*^	−6.56^***^	1.28
Per1		−3.89^**^	−2.37^*^	−1.64
Per2		−3.68^**^	−2.19^*^	−1.68
Npas2		−3.14^**^	−2.89^**^	−1.09
Per3		−2.25	−3.15^*^	1.40
Ucp3		−2.21^*^	−1.53^*^	−1.44
Clock		−2.34	−1.61^**^	−1.45
Arrb1		−1.63^*^	−1.38^*^	−1.19
Rora		−1.01	−1.29	1.28
*Upregulated*				
Dbp		11.12	8.14^**^	1.37
Bhlhe40		2.30^*^	2.18^**^	1.06
Bhlhe41		2.66^*^	2.42^***^	1.10
Cry1		1.74	1.70	1.03
Cry2		2.01	1.62	1.24
Wee1		1.85^*^	2.37^**^	−1.28
Rev-Erbα		3.28	2.31	1.42
**Aging**				
*Upregulated*				
Cd68		2.29^*^	2.10^**^	1.09
Apold1		2.43^**^	2.46^***^	−1.01
Jun		5.41^*^	3.29	1.65
Dusp1		5.84^*^	5.13^*^	1.14
Egr1		17.62^*^	10.94^*^	1.61
**Thyroid differentiation markers**				
Tpo		1.38	−1.37	1.89^*^
Tshr		−1.95	−2.40	1.23
Tg		1.00	1.00	1.00
Thra		−1.18	−1.23	1.04
Thrb		−1.17	−1.28	1.09
Dio1		−1.72	−1.54	−1.12
Dio2		−3.04	−1.18	−2.56
Duox1		−1.22	1.27	−1.55
Duox2		−1.28	−1.58^*^	1.24
Foxe1		−1.28	−2.09^*^	1.63
Glis3		−1.00	−1.80^*^	1.79^*^
Nkx2-1		1.61	−1.30	2.09
Pax8		1.64	−1.90	3.09
Slc5a5		1.06	−2.84	3.01
Slc5a8		−2.40	−1.01	−2.37
**Immunity and inflammation**				
C4b		2.16^*^	2.151^*^	1.01
Lag3		2.20	2.570^*^	−1.17
Clec7a		2.21^*^	2.17^*^	1.02
Mcl1		2.23^*^	2.48^*^	−1.11
Ifi205		2.25	2.56^*^	−1.14
Cd14		2.39	3.75^*^	−1.57
Cd83		2.55	3.17^*^	−1.24
Ccl7		3.56	4.03^*^	−1.13
Nfkbiz		3.75^*^	2.71^**^	1.38
Ccl8		4.65^*^	2.57	1.81
Ccl2		4.74^*^	3.18^*^	1.49
Il1b		5.64	5.14^**^	1.10
Cxcl1		7.67^**^	5.06^*^	1.52
**Obesity development**				
*Downregulated*				
Pcsk1		−5.37^*^	−3.04	−1.77
Cnr1		−3.89^**^	−2.37^*^	−1.64
Scg3		−3.812^*^	−2.36	−1.62^*^
Bbs10		−2.05^**^	−1.53^*^	−1.34^*^
**Lipid metabolism**				
*Downregulated*				
Acot1		−4.67^*^	−2.83^*^	−1.65^**^
Acot2		−2.84^**^	−2.12^**^	−1.34^*^
Tmem171		−4.01	−2.97^**^	−1.35
*Upregulated*				
Ptgs2		5.29^*^	5.14^*^	1.03
**Tumorigenesis and poor prognosis**				
*Upregulated*				
Fhl2		2.01	2.13^*^	−1.06
Epha2		2.40^*^	2.47^*^	−1.03
Apold1		2.43^**^	2.46^***^	−1.01
Tnn		2.74^*^	2.56^*^	1.07
Bhlhe40		2.30^*^	2.18^**^	1.06
Trib1		3.02	2.59^**^	1.17
Mthfd1l		3.07^**^	2.58^*^	1.19
Ccl7		3.56	4.03^*^	−1.13
Ier3		3.83	3.29^***^	1.16
Gprc5a		4.28^**^	2.36^*^	1.81
Ptgs2		5.29^*^	5.14^*^	1.03
Egr1		17.62^*^	10.94^*^	1.61
**Cancer invasion and metastasis**				
*Upregulated*				
Tspan4		2.47^*^	3.29^**^	−1.33
Arl4c		3.05^*^	2.02	1.51
Hspa1a		3.76^*^	2.47	1.53
Hbegf		5.29^*^	7.33^*^	−1.39
Serpine1		7.59^*^	9.22^***^	−1.21
Thbs1		8.75^**^	5.76^**^	1.52
Myc		1.74	1.97	−1.13
AP-1	Jun	5.41^*^	3.29	1.65
	Fos	39.96^**^	11.34^*^	3.52^*^
	Fosb	60.38^*^	23.92^*^	2.52^*^
	Atf3	17.18^*^	20.24^*^	−1.18
	Jdp2	1.91	1.88^*^	1.02
**Anti-apoptotic**				
*Upregulated*				
Phlda1		2.64^*^	2.39^*^	1.11
Mcl1		2.23^*^	2.48^*^	−1.11
Bcl2		−1.58	−1.34	−1.17
Bcl2l1		1.49	1.38	1.08
Bcl2l2		1.11	1.01	1.10
**Tumor progression**				
*Upregulated*				
Cxcl1		7.67^**^	5.06^*^	1.52
Thbs1		8.75^**^	5.76^**^	1.52
**Tumor suppression**				
*Upregulated*				
Csrnp1		2.43^*^	3.50^**^	−1.44^*^
Klf6		2.60^*^	4.14^*^	−1.60^*^
Klf2		2.64^*^	2.89^*^	−1.10
Bhlhe41		2.66^*^	2.42^***^	1.10
Klf4		3.23^**^	3.02^**^	1.07
Plk3		3.53^*^	2.92^*^	1.21
Ppp1r15a		4.31^*^	3.30	1.30
**Favorable prognostic parameters**				
*Upregulated*				
Gem		3.19^*^	2.63^**^	1.21
Socs3		4.07^*^	3.66^*^	1.11

Statistical differences are indicated as *p < 0.05, **p < 0.01, ***p < 0.001.

Genes identified from RNA-seq analyses are represented as Volcano plots for 20- *vs*. 3.5-month-old thyroid (Fig. 2B), and 20- *vs*. 10-month-old thyroid (Fig. 2D). The top five upregulated and downregulated genes, as well as clock genes, are indicated by symbol-marked dots. The top five genes with the largest negative or positive standardized mean difference are represented in bar graphs (Fig. 2C, E). The genes presented in Fig. 2C, E are as follows: *Arntl/Bmal1*, *Tekt1*, *Cyr61*, *Atf3*, *Egr1*, *Fos*, and *Fosb*. This demonstrated significant downregulation of the circadian clock gene *Arntl/Bmal1* and significant upregulation of cell proliferation, migration, and tumorigenesis genes *Cyr61*, *Atf3*, *Egr1*, *Fos*, and *Fosb* in thyroids of aged mice.

Based on the RNA sequencing findings, we focused on aging-related changes in thyroid peripheral clock genes. The expression of positive circadian regulators, including *Arntl/Bmal1*, *Npas2*, *Ucp3*, *Clock*, *Arrb1*, *Ppargc1α*, and *Rorα*, were significantly lower in 20-month-old mice than in 10- or 3.5-month-old mice. The negative feedback regulators, *Per1*, *Per2,* and *Per3* were downregulated, whereas *Bhlhe40* and *Wee1* were upregulated in 20-month-old mice compared with 10- or 3.5-month-old mice (Table 1, Supplementary RNA-seq dataset). The circadian entrainment genes involved in the regulation of the circadian rhythm phase were more downregulated in the thyroids of 20-month-old mice than in those of 10- or 3.5-month-old mice (Table 1, Fig. 1E). These findings suggested that aging causes dysregulation of the peripheral circadian clock, with effects on peripheral clock entrainment in the thyroid.

To confirm these results, we first examined whether the murine thyroid functions as an active peripheral circadian oscillator. The core circadian clock genes *Clock*, *Npas2*, *Arntl/Bmal1*, *Period* (*Per1*, *Per2*, *Per3*), and *Cryptochrome* (*Cry1*, *Cry2*) play critical roles in generating peripheral circadian rhythms. FPKM (Fragment per Kilobase of transcript per Million mapped reads) expression levels of circadian clock genes in the livers and thyroids of 3.5-month-old mice are shown in Supplementary Table S2. The liver is considered a representative peripheral clock organ, and its circadian clock has been thoroughly characterized [26]. We did not detect significant differences in overall FPKM expression levels of circadian clock genes between hepatic and thyroid tissues.

The oscillatory expression of the circadian clock genes examined (*Clock*, *Arntl/Bmal1*, *Rev-erbα*, *Per2*, *Per3*, *Cry1*) was measured in the livers and thyroids of 3.5-month-old mice (Fig. 2F, G, H, Supplementary Table S3). A comparison of the oscillation of *Clock* and *Arntl/Bmal1* expression with that of *Period* genes expression revealed that the negative feedback loop of the circadian clock machinery was functional in both tissues. Upregulation of *Clock* and *Arntl/Bmal1* activates transcription of *Per2*, *Per3*, and *Cry1*, and upregulation of *Per2*, *Per3*, and *Cry1* inhibits *Clock* and *Arntl/Bmal1* [4, 5]. Although the rhythmic patterns of clock genes in the thyroid did not mirror those in the liver, the circadian oscillation of clock genes in the thyroid was preserved and active (Fig. 2H). Collectively, these results indicated that the peripheral circadian clock machinery in the murine thyroid was actively preserved and was independent of that in the liver.

### Downregulation of *Per2* and *Per3* in the aged murine thyroid

**Fig. 3:**
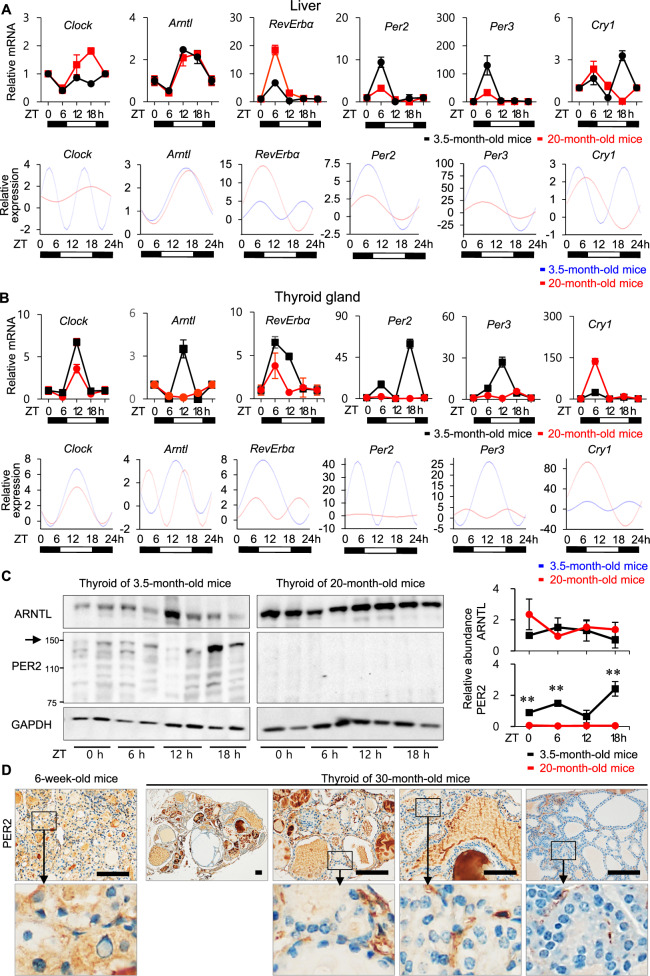
Change of circadian oscillation of peripheral clock genes associated with aging. **A** mRNA expression of *Clock*, *Arntl/Bmal1*, *Rev-erbα*, *Per2*, *Per3*, and *Cry1* in the murine liver of 3.5- and 20-month-old mice at the indicated times. Cosinor-based rhythmometry analysis is used to determine the rhythmic expression of clock genes in the liver of 3.5- and 20-month-old mice. Hepatic oscillation of *Clock*, *Arntl/Bmal1*, *Rev-erbα*, *Per2*, *Per3*, and *Cry1* expression is preserved in 20-month-old mice. **B** mRNA expression of *Clock*, *Arntl/Bmal1*, *Rev-erbα*, *Per2*, *Per3*, and *Cry1* in the murine thyroid of 3.5- and 20-month-old mice at the indicated times. Cosinor-based rhythmometry analysis is used to determine the rhythmic expression of clock genes in the thyroid of 3.5- and 20-month-old mice. Rhythmic expression of *Per2* and *Per3* is significantly lower in the thyroids of 20-month-old mice than in those of 3.5-month-old mice. Each point represents mean ± SEM values of three independent assays (*n* = 3). **C** Immunoblot analysis of thyroids from 3.5- and 20-month-old mice (ZT 0, 6, 12, 18 h; *n* = 2 mice/timepoint) confirms alterations in protein levels of ARNTL/BMAL1 and PER2 consistent with alterations of mRNA levels. GAPDH is used as a loading control. Comparisons are made at individual time points between groups and statistical differences are indicated as *p < 0.05, **p < 0.01, ***p < 0.001. **D** Immunohistochemistry for PER2 in the thyroid of 6-week-old and 30-month-old mice. PER2 is diffusely expressed in the cytoplasm of the follicular epithelium of 6-week-old murine thyroid. The expression of PER2 is very weak in the 30-month-old murine thyroid. Scale bar, 100 μm.

To evaluate further the change of circadian oscillation of peripheral clock genes associated with aging, mature adult (3.5-month-old) and aged (20-month-old) mice were sacrificed every 6 h over the circadian cycle (ZT 0, 6, 12, 18 h). Liver and thyroid tissues were isolated for RT-qPCR at four-time points. First, we investigated how the hepatic circadian rhythm changed with aging. Sato et al. did not detect significant differences in the expression of core clock genes or levels of core clock proteins between young (19–29 weeks) and old mice (55–69 weeks) fed a normal chow diet [27]. Consistently, rhythmic patterns of hepatic circadian clock genes were similar between 3.5- and 20-month-old mice. However, the amplitude of *Clock*, *Per2*, and *Per3* oscillation was significantly decreased in livers of 20-month-old mice relative to 3.5-month-old mice (*p* < 0.05). Therefore, the hepatic oscillation pattern persisted despite decreased amplitude (Fig. 3A). Subsequently, we examined rhythmic expression of circadian clock genes in thyroid tissue. The differences in oscillation between 3.5- and 20-month-old thyroids were significant for *Clock*, *Rev-erbα*, *Per2*, *Per3*, and *Cry1* (*p* < 0.05, Fig. 3B). In thyroids from 20-month-old mice, the expression levels of *Clock*, *Rev-erbα*, *Per2*, and *Per3* were downregulated relative to those in 3.5-month-old mice at every timepoint (ZT 0, 6, 12, 18 h). Peak levels of thyroid *Clock*, *Arntl/Bmal1*, *Rev-erbα*, *Per2*, *Per3*, and *Cry1* decreased dramatically in 20-month-old mice (Fig. 3B). Unlike the robust oscillation *Per2*, and *Per3* in thyroids of 3.5-month-old mice, *Per2*, and *Per3* were not strongly expressed in thyroids of 20-month-old mice, and did not exhibit significant time-specific oscillations (Fig. 3B). In particular, rhythmic expression of *Per2* was markedly blunted in 20-month-old mice.

We measured protein levels of ARNTL/BMAL1 and PER2 in the thyroids of 20- and 3.5-month-old mice across time points (ZT 0, 6, 12, 18 h). The level of PER2 protein was markedly decreased, and the ARNTL/BMAL1 protein level was not significantly different between the thyroids of 20-month-old mice and the thyroids of 3.5-month-old mice (Fig. 3C). In addition, immunohistochemistry for PER2 was performed on the thyroid paraffin block slices of 6-week-old and 30-month-old mice used in our previous study (Supplementary Fig. S3) [11]. In the thyroids of 6-week-old mice, PER2 immunoreactivity was diffusely expressed in the cytoplasm of the follicular epithelium, while the expression of PER2 was very weak in the 30-month-old murine thyroid showing follicular and papillary hyperplasia (Fig. 3D).

Taken together, these findings demonstrated that PER2 protein levels and circadian oscillation of *Per* significantly declined with age in the murine thyroid, suggesting that aging and the accompanying suppression of PER2 expression could contribute to thyroid follicular hyperplasia

### Impaired circadian oscillation of *Per* promotes AP-1 activation in the aged murine thyroid

**Fig. 4:**
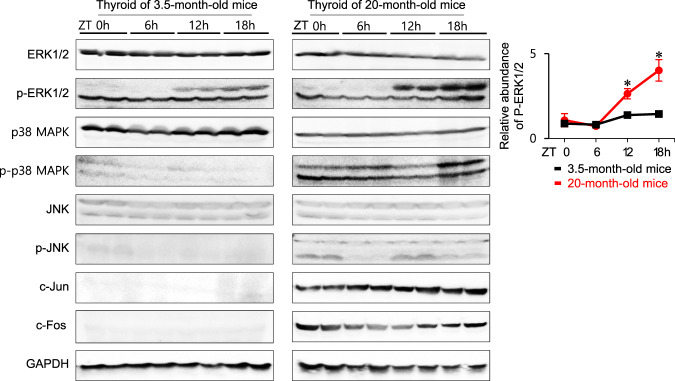
Impaired circadian oscillation of Per promotes AP-1 activation in the aged murine thyroid. Immunoblot analysis of thyroid tissue from 3.5- and 20-month-old mice is used to measure protein levels for the MAPK and PI3K/AKT signaling pathways by timepoint (ZT 0, 6, 12, 18 h). ERK1/2 phosphorylation is higher in the thyroids of 20-month-old mice than in those of 3.5-month-old mice. The graph shows the relative abundances of phospho-ERK1/2 normalized to those of total ERK1/2. The amplitudes of circadian oscillations of phospho-ERK1/2 in the thyroids of 20-month-old mice were larger than those in the thyroids of 3.5-month-old mice. *p < 0.05. Phosphorylation of JNK is higher in the thyroids of 20-month-old mice than in those of 3.5-month-old mice. c-Fos and c-Jun expression is higher in thyroids of 20-month-old mice than in those of 3.5-month-old mice. GAPDH is used as a loading control.

In addition to peripheral circadian clock function, we detected upregulation of AP-1 transcription factor (Fos, Jun, ATF, and JDP) as well as activation of PI3K/AKT and MAPK signaling in transcriptomic analysis of the aged murine thyroid (Table 1, Fig. 2). AP-1 components are important regulators of cell proliferation, differentiation, and apoptosis [28]. In mammalian cells, MAPK cascades include extracellular signal-regulated kinase (ERK), c-Jun amino-terminal kinase (JNK), and the p38 MAPK pathway [29]. MAPK-activated JNK can bind c-Jun, and activated c-Jun upregulates expression of AP-1 target genes, increasing c-Jun and c-Fos protein levels [29]. To determine the effect of aging on key signaling pathways, we measured phosphorylation of ERK, p38 MAPK, JNK, PI3K, AKT, and p70S6 kinase and AP-1 activation in thyroids of 20-month-old mice and 3.5-month-old mice (ZT 0, 6, 12, 18 h) (Fig. 4). Immunoblotting analysis demonstrated diurnal variations in ERK1/2 phosphorylation in the thyroids of 20-month-old mice and 3.5-month-old mice. The amplitudes of circadian oscillations of phospho-ERK1/2 in the thyroids of 20-month-old mice were higher than those in the thyroids of 3.5-month-old mice (Fig. 4, graph). These results demonstrated diurnal variations of ERK phosphorylation in the murine thyroid, which was further enhanced in older thyroid. Phosphorylation of p38 and JNK MAPK was higher in the thyroids of 20-month-old mice than on those of 3.5-month-old mice. While the p38 MAPK appears to play a role, the JNK MAPK is essential for ageing induction of AP-1 in murine thyroid. There were no significant differences in phosphorylation of PI3K/AKT signaling components, including PI3K, AKT, and p70S6 kinase between age groups (data not shown). Protein levels of c-Fos and c-Jun were higher in thyroids of 20-month-old mice than in those of 3.5-month-old mice. Taken together, these results indicated that in aged thyroids, activation of the JNK MAPK pathway increases protein levels of c-Fos and c-Jun.

### Cell proliferation and migration in Nthy-ori 3.1 and TPC1 cells with *PER2* ablation

**Fig. 5:**
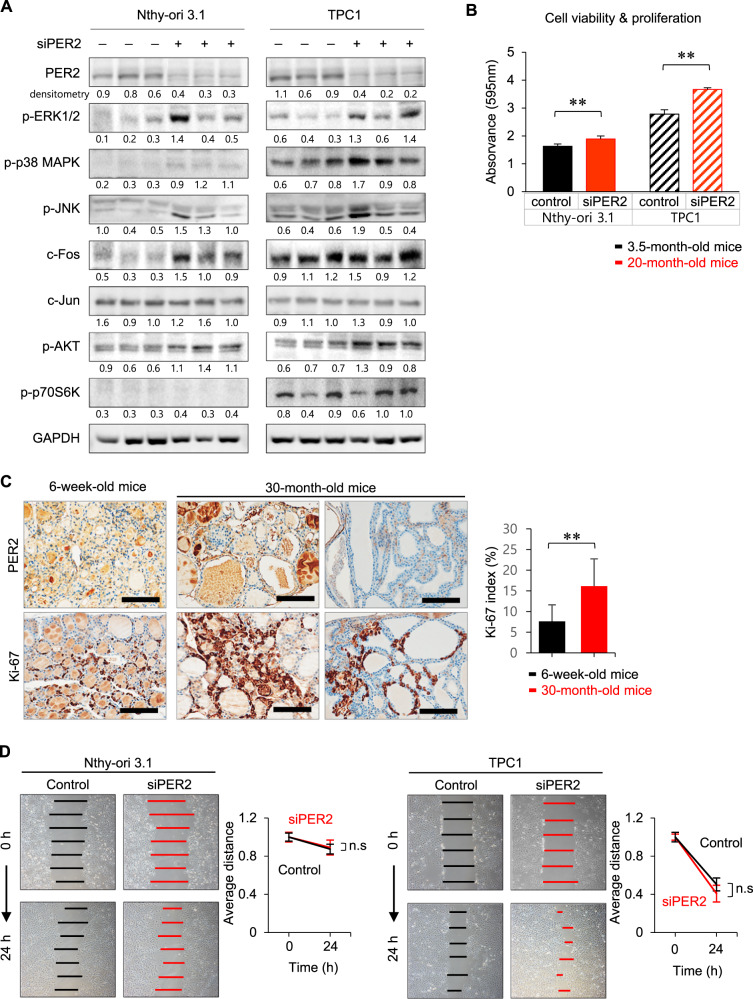
Cell proliferation and migration in Nthy-ori 3.1 and TPC1 cells with PER2 ablation. **A** Immunoblot analysis in Nthy-ori 3.1 and TPC1 cells transfected with si*PER2* or negative control siRNA confirms protein levels for the MAPK pathway and PI3K/AKT signaling pathways. ERK1/2 and JNK MAPK phosphorylation is increased in Nthy-ori 3.1 and TCP1 cells transfected with si*PER2* relative to the corresponding negative control siRNA. Phosphorylation of AKT and p70S6 kinase in the PI3K/AKT signaling pathway is upregulated in Nthy-ori 3.1 and TPC1 cells transfected with si*PER2* relative to the corresponding negative control siRNA. The AP-1 transcription factor c-Fos is upregulated in Nthy-ori 3.1 and TPC1 cells transfected with si*PER2* relative to the corresponding negative control siRNA. No significant difference is detected for c-Jun. GAPDH is used as a loading control. The result of each densitometry analysis is shown below each band and was normalized to the corresponding GAPDH level. **B** Analysis of cell viability and proliferation by MTT assay in control and si*PER2*-transfected cells. Results represent means ± SEM from six independent experiments. *p*-values are obtained by unpaired two-tailed Student’s *t*-test. **C** Cell proliferation, indicated by Ki-67 immunoexpression, is higher in the thyroids of 30-month-old mice with weak PER2 expression (Ki-67 index = 16.12 ± 6.62%) than in that of the thyroids of 6-week-old mice (Ki-67 index = 7.62 ± 4.0%, *p* = 0.004). Scale bar, 100 μm. **p < 0.01. **D** Time-lapse microscopy images of wound closure in control and *PER2* siRNA-transfected Nthy-ori 3.1 and TPC1 cells at 0 and 24 h. The lines define the area lacking cells. Quantification of the wounded width invaded over 24 h by control and *PER2* siRNA-transfected Nthy-ori 3.1 or TPC1 presents as the average distance. Results represent the mean of wound width obtained in two independent experiments consisting of three subsets. n.s, non-significant; *p < 0.05, **p < 0.01.

Having detected declined circadian oscillation of PER2 and activation of AP-1 in aged murine thyroids, we then explored whether impaired circadian oscillation of PER2 affected AP-1 activity and cell proliferation in vitro in human thyroid follicular cells with *PER2* knockdown. *PER2* knockdown increased indicators of MAPK signaling, including phosphorylation of JNK in Nthy-ori 3.1 and TPC1 cells (Fig. 5A). Protein levels of c-Fos in *PER2*-siRNA knockdown Nthy-ori 3.1 and TPC1 cells were increased relative to controls (Fig. 5A). However, c-Jun levels were not significantly changed by *PER2* depletion in Nthy-ori 3.1 or TPC1 cells. There were no significant differences in phosphorylation of AKT or p70S6 kinase (Fig. 5A). These results suggested that *PER2* depletion in thyroid follicular cells induced activation of the AP-1 transcription factor c-Fos via JNK MAPK signaling.

The above result showed that the expression level/ circadian oscillation of PER2 in the murine thyroid with papillary and follicular hyperplasia declined with age. Thus, we next determined if loss of the circadian gene *PER2* affected human thyroid follicular cell viability, proliferation, or migration. MTT assays revealed increased viability and proliferation in Nthy-ori 3.1 cells transfected with si*PER2* (*p* = 0.0037) and TPC1 cells transfected with si*PER2* (*p* = 0.002) relative to the corresponding negative control siRNA for each cell line (Fig. 5B). Furthermore, the Ki-67 proliferation index was increased in the follicular and papillary hyperplasia of 30-month-old murine thyroids (*p* = 0.014, Fig. 5C). The thyroid follicles with a high Ki-67 index corresponded to those with decreased PER2 expression in 30-month-old mice.

The effect of *PER2* knockdown on cell migration was evaluated using a scratch wound healing assay. Migratory potential was not significantly changed in Nthy-ori 3.1 cells transfected with si*PER2* compared with negative control siRNA (*p* = 0.49, Fig. 5D). si*PER2* increased cell migration in TPC1 cells relative to the corresponding negative control siRNA, but this relationship was not statistically significant (*p* = 0.07, Fig. 5D). In the early stages of tumorigenesis, specific oncogene abnormalities are the primary contributors to cell proliferation, but may or may not affect motility/invasion potential [30]. Thus, *PER2* ablation in thyroid cancer could contribute more significantly to processes associated with early stages of tumorigenesis, which involve cell proliferation.

Together, the in vivo and in vitro findings demonstrated that upregulation of the AP-1 transcription factor via JNK MAPK activation in aged murine thyroids is associated with loss of PER2 circadian oscillation. Furthermore, decreases in the overall expression and circadian oscillation of PER2 could increase cell proliferation in the aged thyroid.

### Relationship between PER2 levels and aging in the human thyroid gland

To further interrogate the findings obtained in aged mice and in vitro, we subsequently explored the relationship between aging and potential downregulation of PER2 and subsequent circadian impairment in human thyroid glands (Table 2).

**Table 2:** Relationship between PER2 levels and aging in human thyroid gland samples (A); relationship between PER2 levels and pathological parameters in 90 PTCs (B); relationship between PER2 levels and pathological parameters in 20 PTCs from patients older than 60 years (C).

(A) Non-tumor	Age, years	PER2 (*n*)	*p-*value	PER2 (*n*)	Age, years	*p*-value
	≤60	Negative (42)	0.04	Negative (59)	54 ± 12	0.04
		Positive (28)				
	>60	Negative (17)		Positive (31)	50 ± 11	
		Positive (3)				
(B) Tumor	PER2 (*n*)	Age, years	Tumor size, cm	Multiplicity (*n*)	ETE (*n*)	Central LN meta (*n*)
	Negative (73)	53 ± 12	1.2 ± 0.7	37	56	39
	Positive (17)	47 ± 13	0.9 ± 0.4	3	10	8
	*p*-value	0.04	0.95	0.02	0.14	0.79
(C) Tumor (>60 yr)	PER2 (*n*)	Age, years	Tumor size, cm	Multiplicity (*n*)	ETE (*n*)	Central LN meta (*n*)
	Negative (16)	69 ± 6	1.2 ± 0.8	8	11	14
	Positive (4)	65 ± 6	0.9 ± 0.4	2	3	1
	*p*-value	0.89	0.79	1.00	1.00	0.03

Data are presented as mean ± SEM; little to no expression was considered negative.

*Central LN meta* Central lymph node metastasis.

**Fig. 6:**
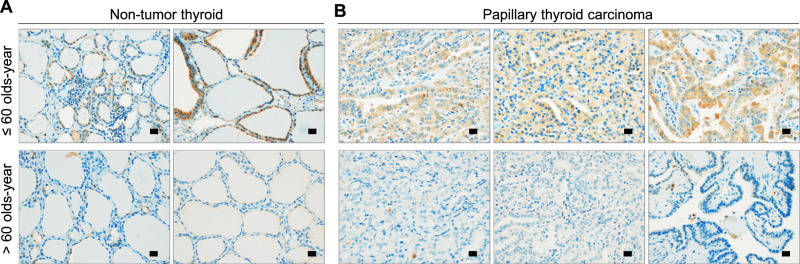
Relationship between PER2 immunoexpression and aging in the human thyroid gland. **A** In non-tumor thyroid, PER2 immunostaining is more frequently observed in the thyroid follicular epithelium of individuals aged <60 years than in those aged >60 years. **B** PER2 is more frequently expressed in PTCs of individuals aged <60 years than in those aged >60 years. Scale bar, 20 μm.

Immunohistochemistry in non-tumor thyroids revealed that PER2 protein levels were significantly reduced in samples over the age of 60 (*p* = 0.04). Thyroids with low expression of PER2 were 54 ± 12 years old and thyroids with high expression of PER2 were 50 ± 11 years old (Table 2A, Fig. 6A, Supplementary Fig. S4). Thus, thyroids with lower expression of PER2 were older than those with high expression of PER2 (*p* = 0.04). We revealed a blunted thyroid circadian rhythm and a decreased protein level of PER2, which were associated with overexpression of AP-1 transcription factors in aged murine thyroids. Overexpression of the AP-1 transcription factor significantly influences tumorigenesis [4, 31]. To determine if decreased circadian oscillation of PER2 is associated with pathological outcomes of patients with PTC, we analyzed the relationship between PER2 expression and pathological parameters in 90 PTCs (Table 2B). Patients with low PER2 expression were older than those with high expression (*p* = 0.04). Lower PER2 levels were significantly associated with tumor multiplicity (*p* = 0.02). In addition, 20 of the samples were collected from PTC patients over 60 years of age (Table 2C). The age of patients with low PER2 expression (69 ± 6 years old) was higher than that of patients with high PER2 expression (65 ± 6 years old), but this relationship was not statistically significant (*p* = 0.89, Fig. 6B, Supplementary Fig. S4). PTCs with lower expression of PER2 were more likely to be accompanied by central lymph node metastasis (*p* = 0.03). There was no significant association between reduced PER2 expression and tumor size (*p* = 0.97), tumor multiplicity (*p* = 1.00), or extrathyroidal extension (ETE, *p* = 1.00). The primary cause of thyroid cancer recurrence is incomplete cervical lymph node dissection or latent cervical lymph node metastases [32]. The incidence of cervical lymph node metastasis for papillary microcarcinoma is comparable to that of PTC [33]. Because it is nearly impossible to detect cervical lymph node micro-metastases by imaging analysis, the incidence of cervical lymph node metastasis is 20–50% even in clinical node-negative patients [32, 34]. Therefore, an index that can predict cervical lymph node metastasis has important potential clinical implications, and decreased PER2 levels in elderly PTC patients could be predictive of cervical lymph node metastasis.

## Discussion

The present study demonstrated the presence of an active peripheral circadian clock in the murine thyroid gland. In young animals, daily oscillations of circadian clock genes in the murine thyroid were similar to those of hepatic circadian rhythms, which is the best characterized independent peripheral circadian clock system. Moreover, using a murine model and human thyroid follicular cell lines, we reported for the first time that aging impacts regulation of the peripheral clock PER2 in the thyroid gland. These aging-related changes did not occur in the liver. Our findings suggest that aging-related attenuation of peripheral thyroid PER2 total levels and circadian rhythmicity contributes to thyroid hyperplasia.

Sellix et al. demonstrated that aging affects the re-entrainment of central and peripheral circadian oscillators in tissue-dependent manners, proposing that this reprogramming could result in age-related declines in the amplitude of circadian clock pacemakers, slowing peripheral circadian re-entrainment and decreasing SCN resistance to external perturbation [35]. The present study revealed significant decrease in circadian entrainment genes with aging, with more linear patterns of *Per2* gene expression during circadian oscillation, and overall decreased levels of PER2 protein. Thus, these peripheral circadian clock changes in thyroid glands from older mice could be related to age-related declines in SCN outputs. TTFL is an autoregulatory negative feedback loop used to describe circadian rhythms in behavior and physiology. The disruption of TTFL results in loss of circadian clock rhythmicity. In this study, we found that, despite the low expression of PER2, a negative regulator, in the thyroids of aged mice, the expression of the positive regulators CLOCK, BMAL1, and Rev-Erbα was downregulated. Therefore, aging could be the cause of changes in circadian rhythmicity mediated by disruption or dysregulation of TTFL in the murine thyroid.

In older humans and rodents, master clock gene expression and circadian oscillation are species- and tissue-dependent. For example, Chen et al. reported that *PER* and *CRY1* are downregulated in orbitofrontal cortex of humans over 60 years of age [36]. Similarly, circadian oscillation of Per1 was absent in lung tissue of aged rats [7]. Age-associated reductions of overall *Clock* and *Bmal1* expression were detected in hamster and mouse SCN [37]. In contrast to downregulated *Bmal1* observed in nearly all brain regions, *Per2* expression was significantly lower only in the dorsomedial hypothalamus and the piriform cortex of aged hamsters [37]. Thus, aging appears to differentially affect circadian clock gene expression in various mammalian species and tissues. In the murine thyroid, the circadian rhythm of PER2/3 is especially affected by aging.

Aging-related histological changes in follicles of aged murine and human thyroids are very similar, including papillary and follicular hyperplasia and irregularly dilated follicles (Supplementary Fig. S3). Compared with younger groups, no significant differences in serum TSH levels were detected in aged mice or humans [11], while other studies have documented the occurrence of hypothyroidism, with higher TSH in relation to aging [38]. It is therefore difficult to conclude that the hyperplastic changes of thyroid follicular cells are due to thyroid dysfunction identified by high TSH levels. The molecular profiles from our RNA sequencing analysis and other independent studies revealed that aging contributes to changes in central circadian clock rhythmicity and localized changes in thyroid metabolism, mitochondrial function, inflammation, immunity, and tumorigenesis in humans and mice [12]. However, among prior findings, the effect of aging on the peripheral circadian clock of thyroid follicles was previously unknown. It is presently unclear whether age-related changes to the thyroid circadian clock is a consequence of the normal aging process. Nevertheless, the impact of thyroid circadian disruption is a potentially significant component of the aging process because the interaction between circadian clock genes and the aging process is bidirectional in that not only do clock gene expression patterns change with age, but also that these changes can accelerate aging [39]. In addition, genetic or functional disruption of molecular clocks can cause genomic instability and accelerate cell proliferation, leading to carcinogenesis [39].

Tissue-specific dysregulation of the circadian machinery considerably alters cellular physiological processes, such as cell cycle regulation, cell proliferation, and metabolism [40]. Moreover, prior studies have reported a correlation between circadian rhythm dysregulation and increased susceptibility to cancer development [8, 9]. Based on these observations, we postulated a potential overlap between aging-related thyroid hyperplasia and thyroid changes associated with loss of PER2 peripheral circadian oscillation. Both in vivo and in vitro, PER2 depletion resulted in activation of the JNK MAPK pathway, which in turn upregulated the c-Fos transcription factors, contributing to age-related thyroid hyperplasia. In mouse liver, the circadian oscillation of ERK1/2 phosphorylation is controlled by circadian core clock components [41]. Thus, the circadian oscillations in ERK1/2 phosphorylation were decreased in clock gene knockout mice [41]. The amplitudes of phospho-ERK1/2 circadian oscillations in the thyroids of aged mice were larger than those in the thyroids of young mice, despite reduced expression of Period genes in the aged mice. Our in vitro study also demonstrated that the phospho-ERK1/2 level was higher in cells transfected with siPER2 than in cells transfected with negative control siRNA. These results suggest that the ERK MAPK pathway is activated with aging in the murine thyroid, but that its activation is independent of the circadian clock machinery of the liver. The present study identified a link between aging-induced thyroid circadian decline of PER2 and thyroid hyperplasia. Future studies using mice with a thyroid-specific deletion of Per2 will be required to clarify further the regulatory mechanisms of the distinct histological changes that occur in the thyroid of aged mice.

Thyroid nodules are more frequent in the elderly, and multinodularity and number of nodules per thyroid also increase with age [42]. Approximately half of adults over the age of 60 years in the United States have thyroid nodules [43]. According to a large-scale multicenter study in Korea, the prevalence of thyroid nodules detected by ultrasonography during medical examination was 34% and increased to 55% in patients aged 70 years or older [44]. Nevertheless, thyroid nodules in elderly have a low risk of malignancy. Since thyroid management in the elderly has an increased risk for multimorbidity, functional and cognitive decline, and treatment complications, accurate assessments of thyroid nodules in the elderly are very important, such that cases requiring intervention can be more easily identified. Tiphaine Mannic et al. demonstrated that thyroid tumors with relatively high cellular differentiation, including follicular and papillary carcinomas, exhibit clock gene oscillations similar to those of normal thyroid and benign thyroid nodules, whereas thyroid carcinomas with decreased cell differentiation exhibit altered circadian oscillations [17]. Xudan Lou et al. identified that expression levels of *CLOCK* and *BMAL1*, positive feedback loop components, are significantly higher in thyroid malignancies than in benign and normal thyroid tissue, while *CRY2*, a component of negative feedback regulation, is significantly downregulated [45]. Therefore, close observation of thyroid nodule clock gene expression and oscillation, especially those of PER2, could be a valuable prognostic indicator in thyroid nodules of elderly patients.

In conclusion, our study not only revealed that thyroid peripheral clock function regulated by PER2 declines with age, but also that thyroid hyperplasia is linked to loss of PER2 circadian oscillation in the aged thyroid. The aging-related impairment of the peripheral thyroid circadian clock function activates the AP-1 transcription factor via JNK MAPK signaling, and could contribute to thyroid hyperplasia. Therefore, we propose our findings as a potential regulatory mechanism for age-related pathologies of the thyroid gland.

## Supplementary information

Reproducibility Checklist

Supplementary Information

Supple Fig. S1A

Supple Fig. S1B

Supple Fig. S1C

Supple Fig. S1D

Supple Fig. S2-S4

supple WB Figure

Data Set 1

## Data Availability

All data generated or analyzed during this study are included in this published article (and its supplementary information files).
