# Supplementary material for: Loss of thyroid gland circadian PER2 rhythmicity in aged mice and its potential association with thyroid cancer development
Source: Cell Death Dis. 2022 Oct 26;13(10):898. doi: 10.1038/s41419-022-05342-2 (PMC9596494; doi:10.1038/s41419-022-05342-2)
Supplement: Supplementary file 2 — Supplementary Information [file 41419_2022_5342_MOESM2_ESM.docx]

**Supplementary Information**

**Loss of thyroid gland circadian PER2 rhythmicity in aged mice and its potential association with thyroid cancer development**

Junguee Lee^1^*, Hae Joung Sul^1^, Hyunsu Choi^2^, Dong Hyun Oh^3^, Minho Shong^4^*

^1^Department of Pathology, Daejeon St. Mary’s Hospital, College of Medicine, The Catholic University of Korea, Seoul, Republic of Korea

^2^Clinical Research Institute, Daejeon St. Mary’s Hospital, College of Medicine, The Catholic University of Korea, Daejeon, Republic of Korea

^3^Department of Radiology, Konyang University Hospital, Daejeon, Republic of Korea

^4^Department of Internal Medicine, Chungnam National University School of Medicine, Daejeon, Republic of Korea

*Correspondance:

Junguee Lee

(junguee@catholic.ac.kr)

Minho Shong

(minhos@cnu.ac.kr)

**SUPPLEMENTARY TABLES & FIGURE LEGENDS**

**Supplementary Table S1.** The sequences of qPCR primers (mouse).

| Genes | Primer forward sequences | Primer reverse sequences |
| --- | --- | --- |
| *Clock* | atgccacagaacagtaccca | ttgtgtggcgaaggtaggat |
| *Arntl/Bmal1* | cactgactaccaagaaagtatg | atccatctgctgccctgaga |
| *RevErbα* | agtcgctgacactacacagg | ccaggtggtgaaggtatctcc |
| *Per2* | cagactcatgatgacagagg | gagatgtacaggatcttccc |
| *Per3* | ccctgtctgtcctctgttgt | ctctctcctttggctggtga |
| *Cry1* | cactggttccgaaagggactc | ctgaagcaaaaatcgccacct |
| *Gapdh* | acagccgcatcttcttgtgcagtg | ggccttgactgtgccgttgaattt |

**Supplementary Table S2.** FPKM expression values of core circadian clock genes in liver and thyroid of 3.5-month-old mice.

| Gene | | *Clock* | *Arntl/Bmal1* | *Per2* | *Per3* | *Cry1* |
| --- | --- | --- | --- | --- | --- | --- |
| Tissue | ZT | FPKM | FPKM | FPKM | FPKM | FPKM |
| Liver | 0 h | 33.64 ± 1.26 | 36.71 ± 0.23 | 39.09 ± 1.56 | 46.41 ± 1.02 | 31.99 ± 0.22 |
|  | 6 h | 34.11 ± 0.34 | 35.28 ± 0.32 | 40.13 ± 3.52 | 40.80 ± 1.99 | 34.04 ± 0.44 |
|  | 12 h | 36.72 ± 0.29 | 35.81 ± 1.15 | 45.39 ± 1.81 | 44.18 ± 1.37 | 35.03 ± 0.22 |
|  | 18 h | 35.83 ± 1.64 | 37.05 ± 0.53 | 42.78 ± 3.25 | 44.74 ± 0.94 | 32.65 ± 0.17 |
| Thyroid | 0 h | 36.94 ± 1.73 | 38.04 ± 0.32 | 40.23 ± 4.08 | 44.11 ± 1.21 | 33.93 ± 0.78 |
|  | 6 h | 40.21 ± 4.61 | 36.18 ± 0.03 | 46.03 ± 1.32 | 41.80 ± 4.36 | 33.11 ± 0.92 |
|  | 12 h | 37.13 ± 0.73 | 35.26 ± 0.34 | 37.99 ± 0.60 | 43.85 ± 1.68 | 36.08 ± 2.70 |
|  | 18 h | 45.02 ± 2.78 | 36.88 ± 1.15 | 44.26 ± 3.17 | 43.29 ± 2.42 | 34.22 ± 1.02 |

FPKM data are presented as mean ± SD. Abbreviations: FPKM, Fragment per kilobase of transcript per million mapped reads; ZT, Zeitgeber time.

**Supplementary Table S3.** Cosinor analysis parameters defining the gene expression rhythms in liver and thyroid of 3.5-month-old and 20-month-old mice.

**Supplementary Fig. S1** Hierarchical clustering heatmap of differential gene expression. **A** The 756 significant genes that met the arbitrary criterium of a ≥ 2-fold change in expression with a raw p-value < 0.05 in Group 1, Group 2, and Group 3. **B** The 445 significant genes that met the arbitrary criterium of a ≥ 2-fold change in expression with a raw p-value < 0.05 in Group 1 and Group 3. **C** The 160 significant genes that met the arbitrary criterium of a ≥ 2-fold change in expression with a raw p-value < 0.05 in Group 2 and Group 3. **D** The 340 significant genes that met the arbitrary criterium of a ≥ 2-fold change in expression with a raw p-value < 0.05 in Group 1 and Group 2.

**Supplementary Fig. S2** Cosinor-based rhythmometry analysis of peripheral circadian clocks in liver and thyroid of 3.5-month-old and 20-month-old mice.

**Supplementary Fig. S3** Aging-related histological changes in the thyroid follicles of the aged murine. The thyroid gland of 6-week-old mice was composed of variable-sized follicles with a relatively round shape. The thyroid glands from 30-month-old mice were composed of irregularly dilated follicles and follicles showing papillary and glandular hyperplasia.

**Supplementary Fig. S4** PER2 immunohistochemical staining of tissue microarrays of the human thyroid gland.

**Supplementary RNAseq dataset.** Excel file showing the expression of the 15,349 genes in the thyroids of 20-month-old, 10-month-old and 3.5-month-old mice.
