# Supplementary figures and images for: Loss of thyroid gland circadian PER2 rhythmicity in aged mice and its potential association with thyroid cancer development
Source: Cell Death Dis. 2022 Oct 26;13(10):898. doi: 10.1038/s41419-022-05342-2 (PMC9596494; doi:10.1038/s41419-022-05342-2)

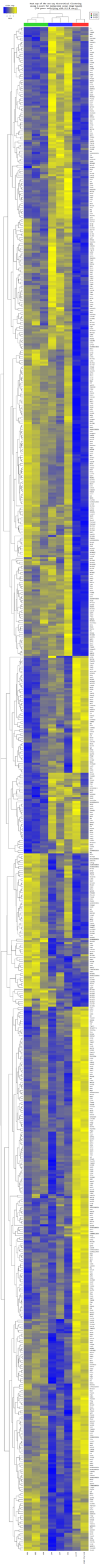

Supplement: Supplementary file 3 — Supple Fig. S1A [file 41419_2022_5342_MOESM3_ESM.png]

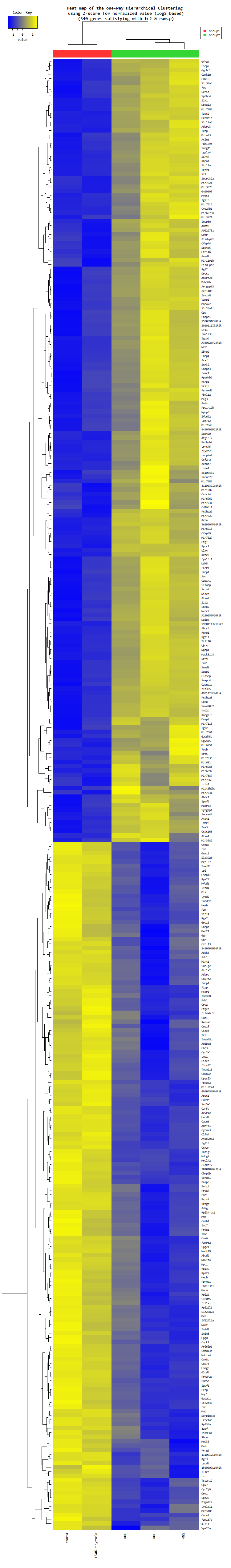

Supplement: Supplementary file 4 — Supple Fig. S1B [file 41419_2022_5342_MOESM4_ESM.png]

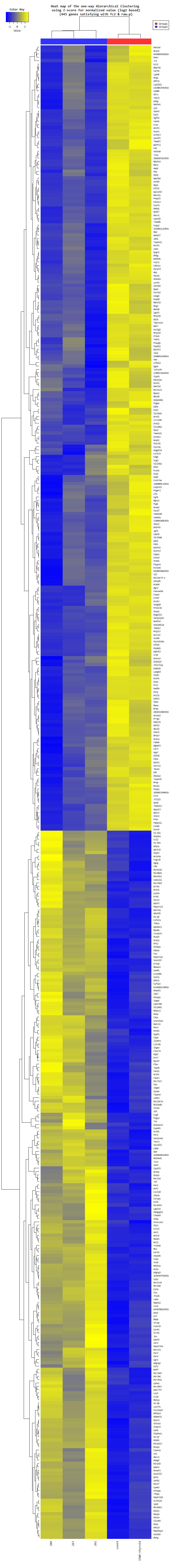

Supplement: Supplementary file 5 — Supple Fig. S1C [file 41419_2022_5342_MOESM5_ESM.png]

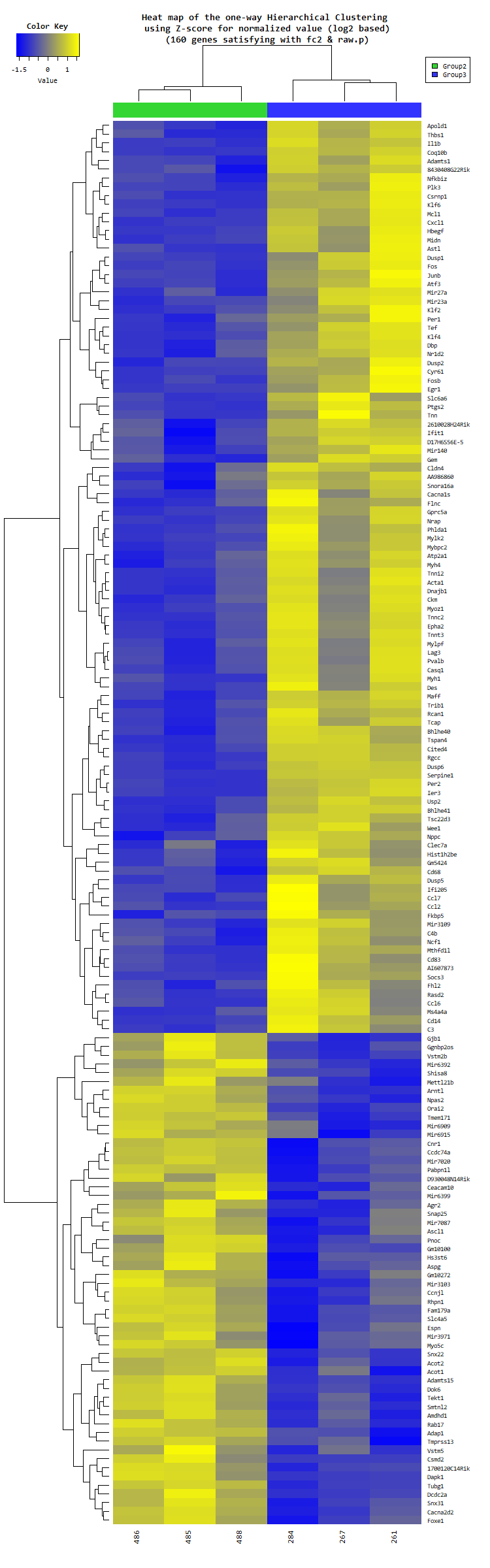

Supplement: Supplementary file 6 — Supple Fig. S1D [file 41419_2022_5342_MOESM6_ESM.png]
