# Supplementary material for: Loss of thyroid gland circadian PER2 rhythmicity in aged mice and its potential association with thyroid cancer development
Source: Cell Death Dis. 2022 Oct 26;13(10):898. doi: 10.1038/s41419-022-05342-2 (PMC9596494; doi:10.1038/s41419-022-05342-2)
Supplement: Supplementary file 7 — Supple Fig. S2-S4 [file 41419_2022_5342_MOESM7_ESM.pptx]

## Slide 1
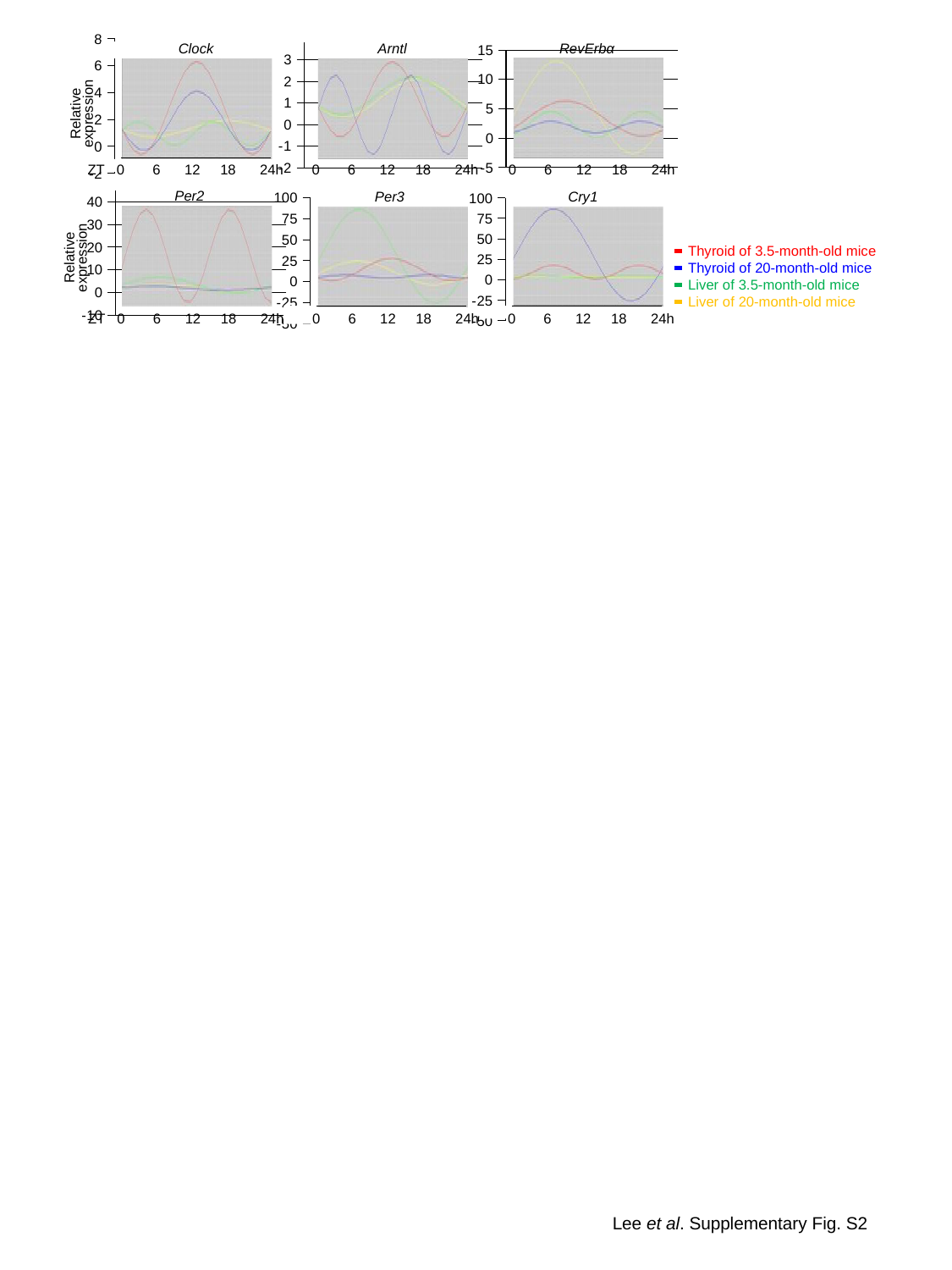

### Chart
| Category | 계열 2 |
|---|---|
| 항목 1 | 2.4 |
| 항목 2 | 4.4 |
| 항목 3 | 1.8 |
| 항목 4 | 2.8 |Clock
ZT 0 6 12 18 24h
RevErbα
### Chart
| Category | 계열 2 |
|---|---|
| 항목 1 | 2.4 |
| 항목 2 | 4.4 |
| 항목 3 | 1.8 |
| 항목 4 | 2.8 |
0 6 12 18 24h
Arntl
### Chart
| Category | 계열 2 |
|---|---|
| 항목 1 | 2.4 |
| 항목 2 | 4.4 |
| 항목 3 | 1.8 |
| 항목 4 | 2.8 |
0 6 12 18 24h
Per2
### Chart
| Category | 계열 2 |
|---|---|
| 항목 1 | 2.4 |
| 항목 2 | 4.4 |
| 항목 3 | 1.8 |
| 항목 4 | 2.8 |
ZT 0 6 12 18 24h
Cry1
### Chart
| Category | 계열 2 |
|---|---|
| 항목 1 | 2.4 |
| 항목 2 | 4.4 |
| 항목 3 | 1.8 |
| 항목 4 | 2.8 |
0 6 12 18 24h
Per3
### Chart
| Category | 계열 2 |
|---|---|
| 항목 1 | 2.4 |
| 항목 2 | 4.4 |
| 항목 3 | 1.8 |
| 항목 4 | 2.8 |
0 6 12 18 24h
Thyroid of 3.5-month-old mice
Thyroid of 20-month-old mice
Liver of 3.5-month-old mice
Liver of 20-month-old mice
Relative expression
Relative expression
Lee et al. Supplementary Fig. S2

## Slide 2
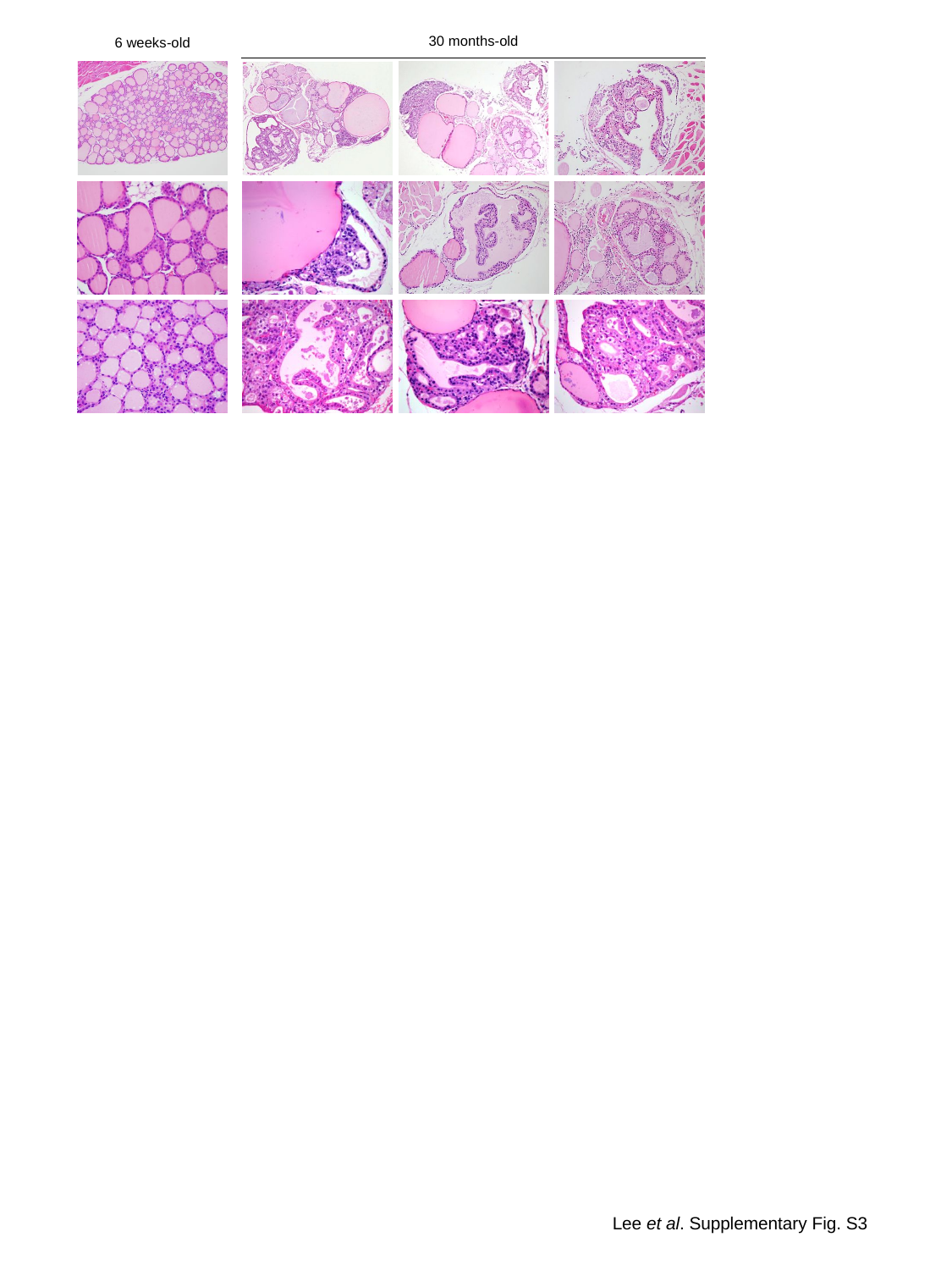

30 months-old
6 weeks-old
Lee et al. Supplementary Fig. S3

## Slide 3
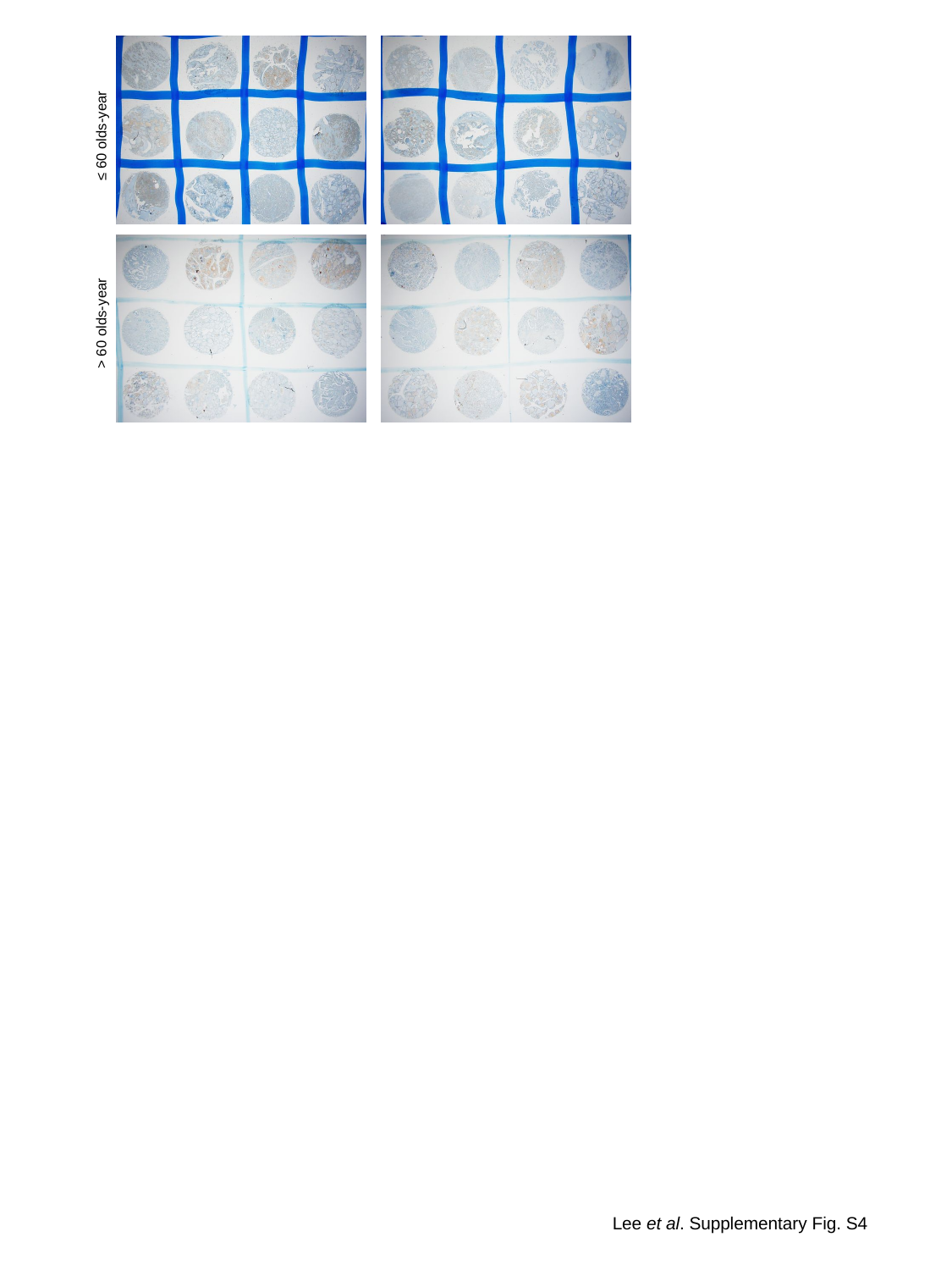

≤ 60 olds-year
> 60 olds-year
Lee et al. Supplementary Fig. S4
