# Supplementary material for: Loss of thyroid gland circadian PER2 rhythmicity in aged mice and its potential association with thyroid cancer development
Source: Cell Death Dis. 2022 Oct 26;13(10):898. doi: 10.1038/s41419-022-05342-2 (PMC9596494; doi:10.1038/s41419-022-05342-2)
Supplement: Supplementary file 8 — supple WB Figure [file 41419_2022_5342_MOESM8_ESM.pptx]

## Slide 1
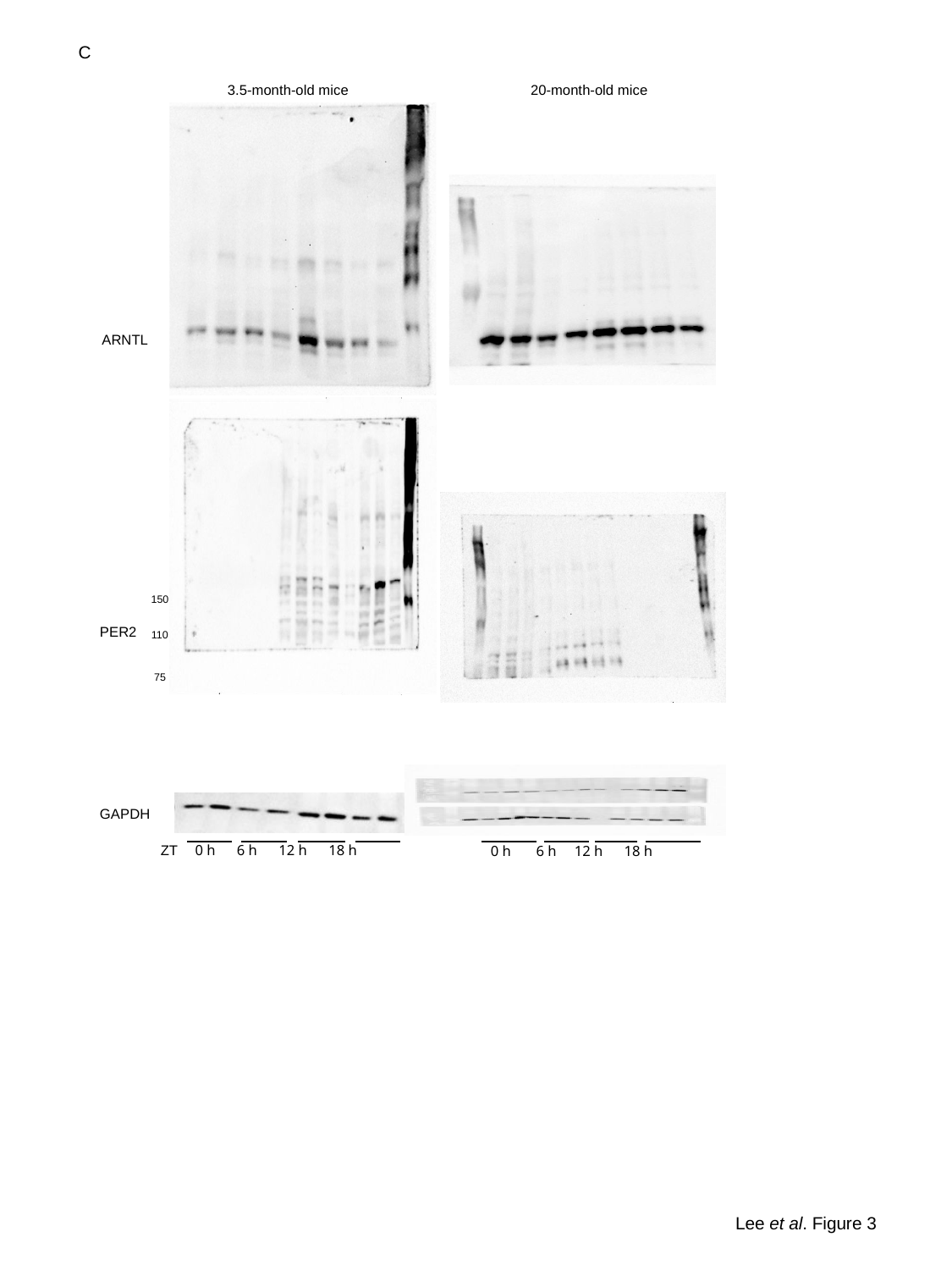

C
3.5-month-old mice
20-month-old mice
ARNTL
150
PER2
110
75
GAPDH
ZT
 0 h 6 h 12 h 18 h
 0 h 6 h 12 h 18 h
Lee et al. Figure 3

## Slide 2
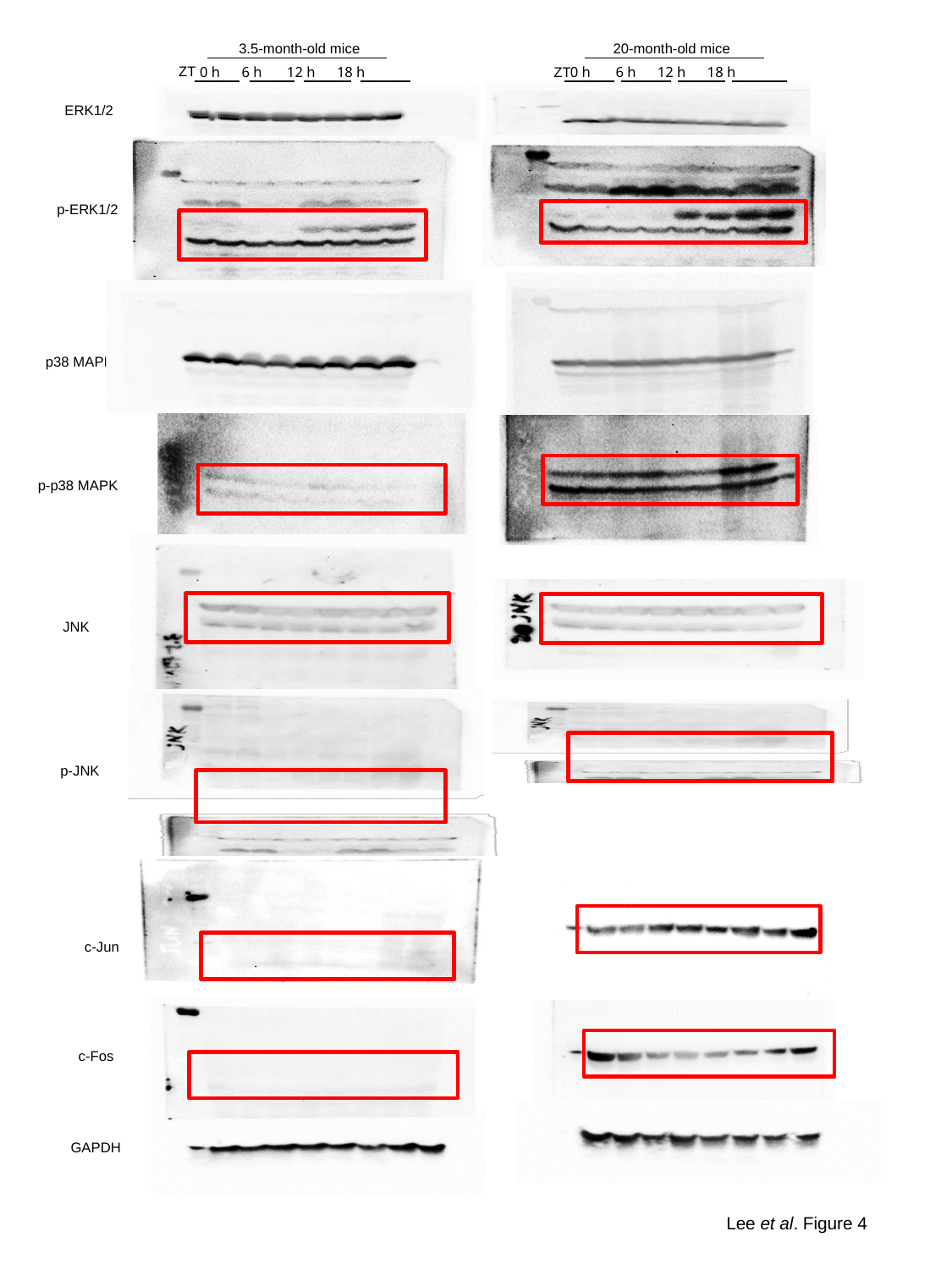

3.5-month-old mice
ZT
 0 h 6 h 12 h 18 h
20-month-old mice
ZT
 0 h 6 h 12 h 18 h
ERK1/2
p-ERK1/2
p38 MAPK
p-p38 MAPK
JNK
p-JNK
c-Jun
c-Fos
GAPDH
Lee et al. Figure 4

## Slide 3
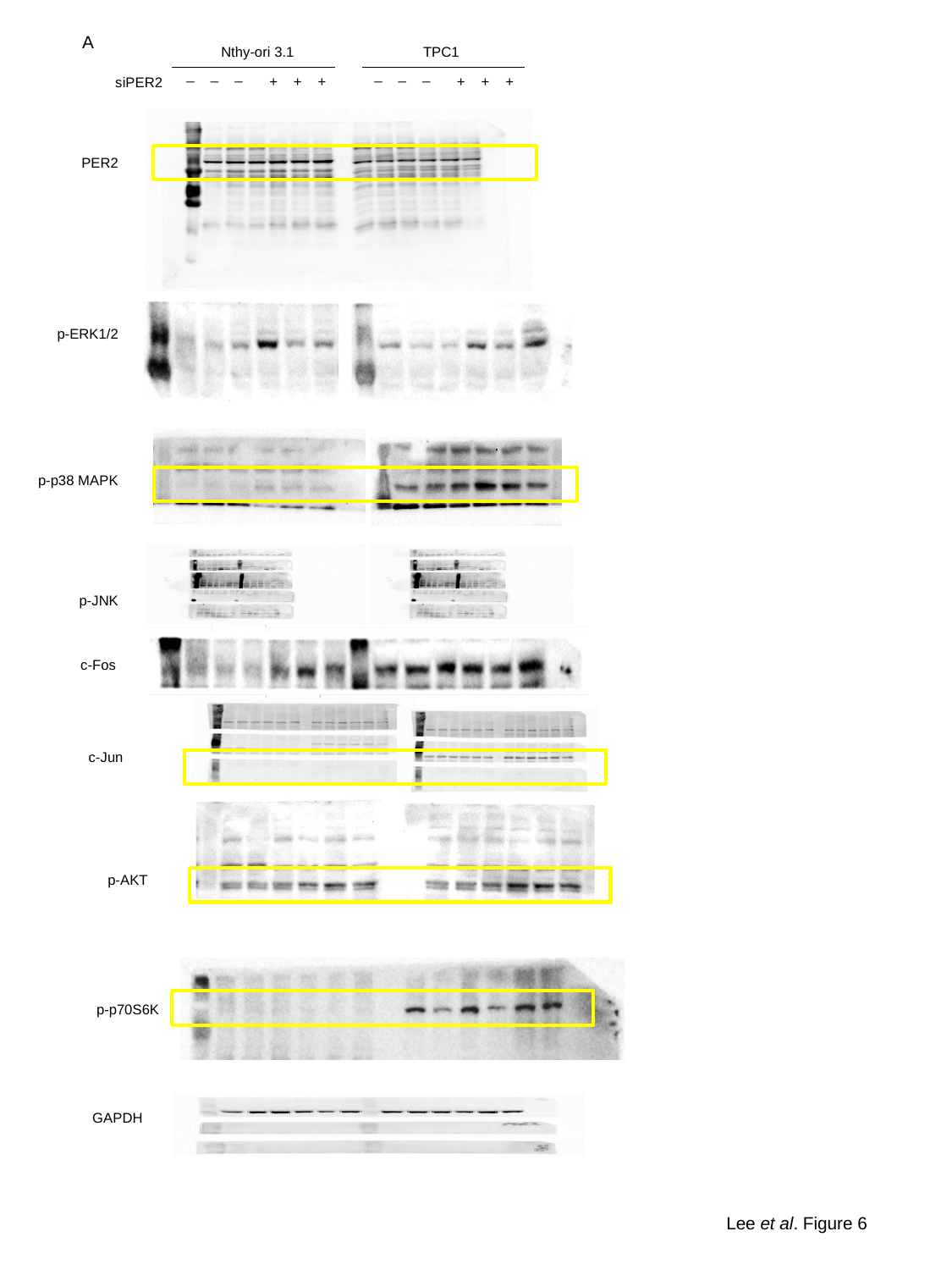

A
Nthy-ori 3.1
TPC1
siPER2
 + + +
­̶ ­̶ ­̶
 + + +
­̶ ­̶ ­̶
PER2
p-ERK1/2
p-p38 MAPK
p-JNK
c-Fos
c-Jun
p-AKT
p-p70S6K
GAPDH
Lee et al. Figure 6
